# Lidocaine Versus Lignocaine: A Scoping Review of Terminological Usage in Dental Local Anesthesia Literature

**DOI:** 10.7759/cureus.115757

**Published:** 2026-09-04

**Authors:** Takutoshi Inoue, Tomoaki Ujita, Toru Yamamoto, Naotaka Kishimoto

**Affiliations:** 1 Department of Anatomy, Teikyo University School of Medicine, Tokyo, JPN; 2 Division of Dental Anesthesiology, Niigata University Medical and Dental Hospital, Niigata, JPN; 3 Division of Dental Anesthesiology, Graduate School of Medicine, Dentistry and Health Sciences, Niigata University, Niigata, JPN

**Keywords:** lidocaine, lignocaine, local anesthesia, scoping review, terminology

## Abstract

Although *lidocaine* is the internationally accepted International Nonproprietary Name (INN), the alternative term *lignocaine* continues to be used in English-language dental literature. However, patterns and consistency of terminology usage within individual publications have not been systematically investigated. This scoping review systematically mapped terminology usage in 526 English-language dental articles related to local anesthetics or local anesthesia techniques identified through searches of PubMed and Web of Science. Articles were classified into three groups according to terminology usage: Lidocaine (Lido), Lignocaine (Ligno), and Lidocaine+Lignocaine (Lido+Ligno). Terminology usage was compared between the Title/Abstract/Keywords (TAK) and Full Text (FT), and a proportion difference was calculated to evaluate differences across dental specialties and geographic regions. The Lido group predominated in both TAK and FT, whereas the proportion of the Lido+Ligno group increased in FT. The proportion difference was negative for the Lido and Ligno groups but positive for the Lido+Ligno group. Asia showed the largest differences between TAK and FT, with distinctive terminology patterns observed in India, whereas terminology usage remained relatively consistent in Japan, Korea, and Taiwan. Although lidocaine remains the predominant spelling in English-language dental literature, concurrent use of lidocaine and lignocaine within individual publications is common. These findings highlight the importance of consistent terminology in dental academic communication. The analytical framework presented in this study may also be applicable to investigations of other synonymous medical and dental terms.

## Introduction and background

Since its introduction in 1943, lidocaine has been one of the most widely used local anesthetics for pain control in dental treatment and surgical procedures [1]. In dentistry, it is administered through various techniques, including infiltration anesthesia, nerve block anesthesia, topical anesthesia, and iontophoresis [2,3]. Although articaine, which is widely used worldwide, has recently become commercially available in Japanese dental practice [4], lidocaine remains a cornerstone local anesthetic in dental anesthesia.

Despite denoting the same chemical entity, two English spellings, “lidocaine” and “lignocaine”, coexist as synonymous terms in dental academic literature. Historically, “lignocaine” was used predominantly in the United Kingdom, whereas “lidocaine” was subsequently adopted as the Recommended International Nonproprietary Name (INN) for this local anesthetic [5]. The choice between these terms may reflect regional differences and disciplinary conventions. Indeed, both spellings are sometimes used within a single publication, indicating inconsistency in terminology usage.

Such terminological inconsistency may affect scientific communication and terminology consistency in the dental literature [6]. Whether it influences literature retrieval or database indexing requires further investigation. Furthermore, such variability may confuse early-career researchers and non-specialists and may hinder efficient international academic communication.

A substantial body of basic and clinical research on local anesthetics, including lidocaine, has accumulated in dentistry. Although a few studies have systematically investigated terminology usage in medical and anatomical literature, comparable analyses focusing on the synonymous terms “lidocaine” and “lignocaine” in dental local anesthesia literature have not been reported. In particular, no comprehensive analysis has compared spelling differences across structured bibliographic components - titles, abstracts, keywords, and full text - or across geographic regions.

The present scoping review aimed to systematically map and characterize the usage patterns of the English spellings “lidocaine” and “lignocaine” in the dental literature by comparing terminology across titles, abstracts, keywords, and full text, as well as across specialties, geographic regions, and time periods.

## Review

Methods

Methodological Framework

A preliminary assessment confirmed that, in dental academic literature, the same chemical entity, lidocaine, is represented by two alternative spellings, “lidocaine” and “lignocaine,” and that both forms may coexist within a single publication. On the basis of these observations, the methodological framework proposed by Arksey and O’Malley was adopted to ensure a structured and transparent approach to the literature review [7]. This framework comprises the following sequential stages: (1) identification of the research question; (2) comprehensive identification of relevant studies; (3) study selection; (4) data charting; (5) collation and summarization of the results; and (6) reporting of the findings. It is particularly suitable for mapping terminology usage patterns within a specific field. Unlike bibliometric analyses, which primarily quantify publication characteristics and citation relationships, this study aimed to systematically identify and characterize terminology usage within individual publications. Therefore, the scoping review framework proposed by Arksey and O'Malley was considered more appropriate for addressing the present research question. This study was conducted as a literature-based scoping review of publicly available dental literature. Because it did not involve human participants or identifiable personal data, institutional review board approval was not required.

Study Design

This study was designed as a literature-based scoping review to systematically characterize the usage patterns of the English spellings “lidocaine” and “lignocaine” for the local anesthetic lidocaine in dental academic literature. The included articles were classified into three groups according to terminology usage: the Lidocaine group (Lido group), the Lignocaine group (Ligno group), and the combined group (Lido+Ligno group), in which both spellings were used within the same publication. The primary research objectives were as follows: (1) to analyze the usage patterns and proportion difference of each group across the title, abstract, keywords, and full text; (2) to compare usage patterns by specialty and geographic region; and (3) to identify regions in which “lignocaine” is predominantly used as the preferred English spelling in dental literature.

Search Strategy

This scoping review was conducted to systematically identify and characterize the usage patterns of the English spellings of lidocaine (“lidocaine” and “lignocaine”) in dental academic literature. This scoping review was conducted according to the methodological framework proposed by Arksey and O’Malley [7] and reported in accordance with the Preferred Reporting Items for Systematic Reviews and Meta-Analyses Extension for Scoping Reviews (PRISMA-ScR) guidelines [8]. English-language dental articles published online on or before November 29, 2025, were eligible for inclusion. The final literature search was conducted on November 29, 2025, using PubMed and Web of Science. These databases were selected because they provide broad and complementary coverage of biomedical, dental, and multidisciplinary literature. The search yielded a sufficiently large body of literature for mapping terminology usage in the present scoping review; therefore, it was not extended to additional databases. The term “dental” was used to define a focused corpus of dental literature because the objective of this review was to map the usage of “lidocaine” and “lignocaine” specifically within dental academic literature. The search strategy is presented in Table 1. Records retrieved from each database were imported into EndNote Basic (Clarivate) for integration and deduplication. After duplicate removal, the remaining records were organized and managed in Microsoft 365^®^ Excel. The review was designed to identify terminology usage rather than to synthesize clinical evidence.

**Table 1:** Search strategy used in this scoping review

Databases	Search query	Publication period	Language
PubMed and Web of Science	(“lidocaine” OR “lignocaine”) AND “dental”	Up to November 29, 2025	English

Study Selection

Study selection was conducted in two stages: an initial screening of titles and abstracts, followed by a full-text eligibility assessment. Two reviewers (T.I. and T.U.) independently performed both stages of screening. In cases of disagreement, consensus was reached through discussion with a third reviewer (T.Y.). Final inclusion decisions were made through discussion among the research team under the supervision of a senior author (N.K.).

Eligibility Criteria

This study included full-text, peer-reviewed English-language articles related to lidocaine in the field of dentistry. Eligible studies addressed local anesthetics or local anesthesia techniques. These eligibility criteria were established to focus the review on English terminology usage in peer-reviewed dental literature specifically addressing the local anesthetic use of lidocaine. To enable direct comparison of terminology usage between bibliographic information and the corresponding full text within the same publication, articles were required to contain at least one of the two spellings (“lidocaine” or “lignocaine”) in both Title/Abstract/Keywords (TAK) and Full Text (FT). The detailed inclusion and exclusion criteria are presented in Table 2.

**Table 2:** Inclusion and exclusion criteria for study selection

Inclusion criteria	Exclusion criteria
Full-text articles	Studies focusing on routes of administration other than local administration
Peer-reviewed articles	Articles in which “lidocaine” and/or “lignocaine” appeared only in TAK or only in FT
Articles published in English	Articles containing neither “lidocaine” nor “lignocaine”
Articles related to lidocaine in the field of dentistry	Articles referring only to brand names (e.g., Xylocaine®)
Articles addressing local anesthetics or local anesthesia techniques, including infiltration anesthesia, nerve block anesthesia, topical anesthesia, and iontophoresis	Case reports
Articles containing “lidocaine” and/or “lignocaine” in both Title/Abstract/Keywords (TAK) and Full Text (FT)	Letters
―	Study protocols
―	Guidelines

Data Extraction and Definitions

For all included articles, the full text (excluding references) was reviewed in detail. The following data were extracted: author names, article title, year of publication, field of specialization, and the country of affiliation of the first author. Fields of specialization were categorized into two groups: Clinical medicine, defined as studies involving human subjects, including clinical research, clinical trials, and clinical evaluations; and Basic science, defined as studies involving in vitro experiments, animal experiments, or histological and cell-biological investigations. For each article, terminology usage was assessed separately in TAK and FT. The presence of the terms “lidocaine,” “lignocaine,” or both was recorded for each section.

Definition of Proportion Difference

In this study, the proportion difference was introduced as an exploratory descriptive indicator to assess differences in terminology usage between bibliographic information (TAK) and the corresponding full text (FT). It was defined as the difference between the proportion of a given group in FT and that in TAK. Because TAK and FT differ in text length and function, the proportion difference is intended to be interpreted as an index of relative differences in terminology selection rather than a direct measure of linguistic frequency. A positive proportion difference indicates that the group was used relatively more frequently in FT than in TAK, whereas a negative value indicates that it was used relatively more frequently in TAK than in FT. Because the classification was performed at the article level rather than by counting individual word occurrences, the proportion difference reflects differences in terminology usage patterns within publications rather than simple differences in word frequency.

Results

Study Selection

A preliminary database search identified 1,771 articles. After duplicate removal, 1,679 articles remained for screening. Following title and abstract screening, 761 articles were excluded on the basis of relevance. Full-text assessment resulted in the inclusion of 526 articles (Figure 1; Supplemental Data). The included articles comprised 462 Original Articles, 61 Review Articles, and 3 Technical Reports.

**Figure 1:**
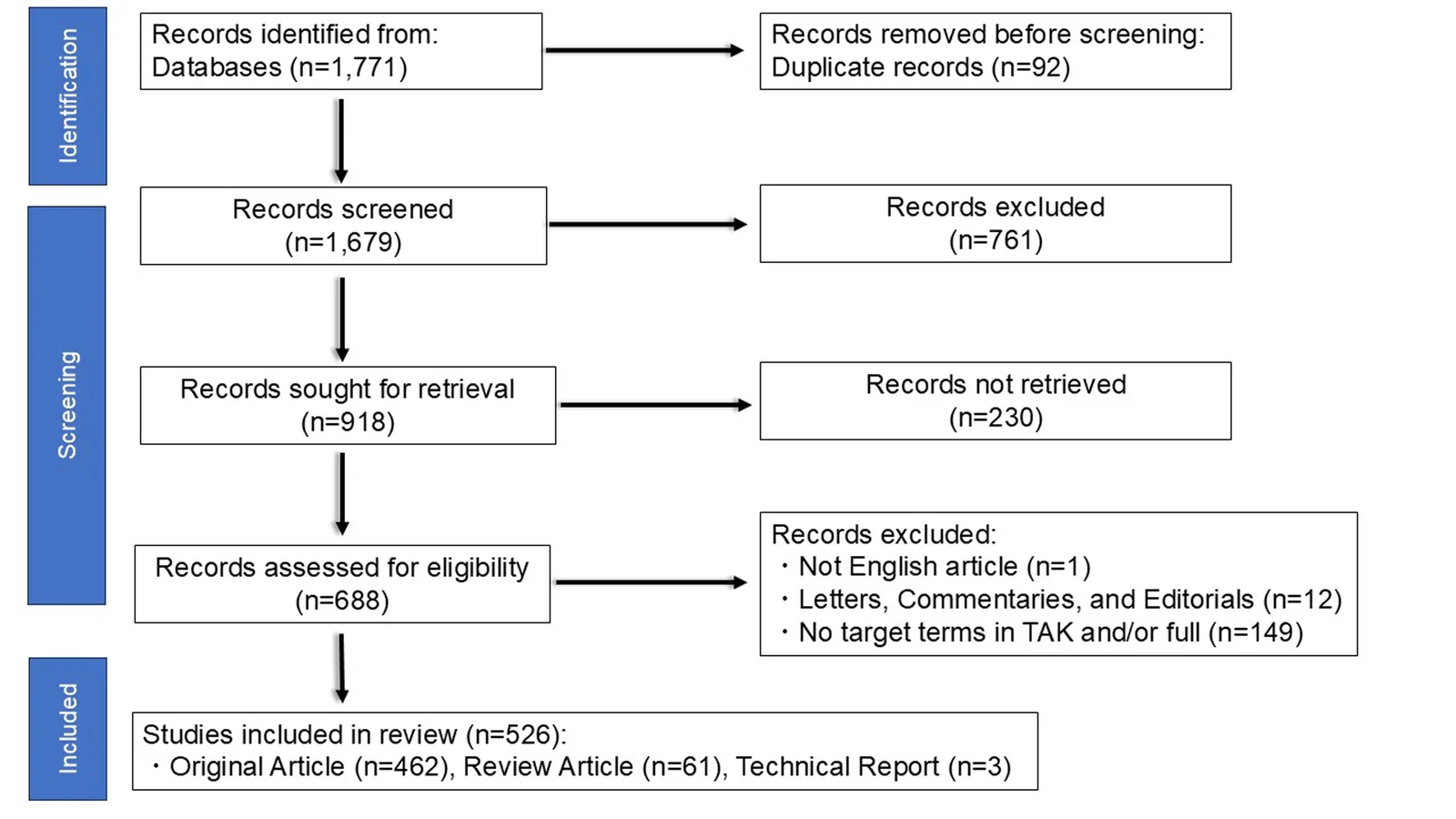
Identification of studies via databases with the PRISMA flow diagram PRISMA: Preferred Reporting Items for Systematic Reviews and Meta-Analyses; TAK: Title/Abstract/Keywords

Overall Results

Across the 526 included English-language articles, terminology usage in TAK showed that the Lido group accounted for 82%, the Ligno group for 14%, and the Lido+Ligno group for 4%. In FT, the corresponding proportions were 75%, 8%, and 17%, respectively (Table 3). In both TAK and FT, the Lido group was the most prevalent, whereas the proportion of the Lido+Ligno group increased from TAK to FT.

**Table 3:** Overall terminology usage in TAK and FT

			Title, Abstract, Keyword (TAK)		Full text (FT)		Proportion difference
Total			526 (100%)		526 (100%)		0%
Overall result		Lido group	431 (82%)		397 (75%)		-7%
		Ligno group	75 (14%)		42 (8%)		-6%
		Lido + Ligno group	20 (4%)		87 (17%)		13%

Analysis of the proportion difference yielded values of −7%, −6%, and 13% for the Lido, Ligno, and Lido+Ligno groups, respectively (Table 3). Negative values were observed for the single-notation groups, whereas a positive value was observed for the combined group.

Specialization

Of the 526 English-language articles, 383 were classified as Clinical medicine and 143 as Basic science, with Clinical medicine accounting for the majority (Table 4).

**Table 4:** Distribution of terminology usage by specialization in TAK and FT

			Title, Abstract, Keyword (TAK)		Full text (FT)		Proportion difference
Total			526 (100%)		526 (100%)		0%
Specialization	Clinical Medicine	Lido group	383 (72.8%)	297 (78%)	383 (72.8%)	264 (69%)	-9%
		Ligno group		69 (18%)		38 (10%)	-8%
		Lido + Ligno group		17 (4%)		81 (21%)	17%
	Basic Science	Lido group	143 (27.2%)	134 (94%)	143 (27.2%)	133 (93%)	-1%
		Ligno group		6 (4%)		4 (3%)	-1%
		Lido + Ligno group		3 (2%)		6 (4%)	2%

In TAK, the Lido group accounted for 78%, the Ligno group for 18%, and the Lido+Ligno group for 4%. In FT, the corresponding proportions were 69%, 10%, and 21%, respectively (Table 4). As in the overall analysis, the Lido group was the most prevalent in both TAK and FT, and the proportion of the Lido+Ligno group increased from TAK to FT.

Analysis of the proportion difference by specialization revealed values of −9%, −8%, and 17% for the Lido, Ligno, and Lido+Ligno groups, respectively, in Clinical medicine. In Basic science, the corresponding values were −1%, −1%, and 2% (Table 4). In both specialties, negative values were observed for the single-notation groups, whereas positive values were observed for the combined group.

Country of Affiliation

Among the 526 English-language articles, the regional distribution of first-author affiliations was as follows: Asia (n = 258), Oceania (n = 10), North America (n = 67), Latin America (n = 42), Europe (n = 77), the Middle East (n = 69), and Africa (n = 3), with Asia accounting for the largest proportion (Table 5).

**Table 5:** Distribution of terminology usage by country of affiliation in TAK and FT

			Title, Abstract, Keyword (TAK)		Full text (FT)		Proportion difference
Total			526 (100%)		526 (100%)		0%
Country of Affiliation	Asia	Lido group	258 (49%)	178 (68%)	258 (49%)	163 (63%)	-5%
		Ligno group		64 (25%)		32 (12%)	-13%
		Lido + Ligno group		16 (6%)		63 (24%)	18%
	Oceania	Lido group	10 (1.9%)	7 (70%)	10 (1.9%)	6 (60%)	-10%
		Ligno group		2 (20%)		2 (20%)	0%
		Lido + Ligno group		1 (10%)		2 (20%)	10%
	North America	Lido group	67 (12.7%)	67 (100%)	67 (12.7%)	61 (91%)	-9%
		Ligno group		0 (0%)		0 (0%)	0%
		Lido + Ligno group		0 (0%)		6 (9%)	9%
	Latin America	Lido group	42 (7.9%)	41 (98%)	42 (7.9%)	39 (93%)	-6%
		Ligno group		1 (2%)		1 (2%)	0%
		Lido + Ligno group		0 (0%)		2 (5%)	5%
	Europe	Lido group	77 (14.6%)	68 (88%)	77 (14.6%)	64 (83%)	-5%
		Ligno group		6 (8%)		6 (8%)	0%
		Lido + Ligno group		3 (4%)		7 (9%)	5%
	Middle East	Lido group	69 (13.1%)	68 (99%)	69 (13.1%)	62 (90%)	-9%
		Ligno group		1 (1%)		0 (0%)	-1%
		Lido + Ligno group		0 (0%)		7 (10%)	10%
	Africa	Lido group	3 (0.5%)	2 (67%)	3 (0.5%)	2 (67%)	0%
		Ligno group		1 (33%)		1 (33%)	0%
		Lido + Ligno group		0 (0%)		0 (0%)	0%

Analysis of terminology usage across regions showed that the Lido group was the most prevalent in all regions in both TAK and FT. Analysis of the proportion difference by region revealed negative values for the single-notation groups and positive values for the combined group in most regions (Table 5).

In Asia, which had the largest number of publications, the absolute values of the proportion difference were greater than those in other regions. Although similar patterns were observed in other regions, the magnitude of change was smaller than that observed in Asia.

Term Use in Asia

Within Asia, the countries represented were India (n = 117), Japan (n = 86), China (n = 16), Korea (n = 16), Pakistan (n = 7), Thailand (n = 6), Taiwan (n = 4), Singapore (n = 4), and Malaysia (n = 2), with India accounting for the largest share (Table 6).

**Table 6:** Terminology usage of “lidocaine” and “lignocaine” in Asia

		Title, Abstract, Keyword (TAK)		Full text		Proportion difference
Number of publications in Asia		258 (100%)		258 (100%)		0%
Japan	Lido group	86 (33.3%)	85 (99%)	86 (33.3%)	85 (99%)	0%
	Ligno group		0 (0%)		0 (0%)	0%
	Lido + Ligno group		1 (1%)		1 (1%)	0%
India	Lido group	117 (45.3%)	44 (38%)	117 (45.3%)	35 (30%)	-8%
	Ligno group		60 (51%)		31 (26%)	-25%
	Lido + Ligno group		13 (11%)		51 (44%)	33%
China	Lido group	16 (6.2%)	15 (94%)	16 (6.2%)	14 (88%)	-6%
	Ligno group		1 (6%)		0 (0%)	-1%
	Lido + Ligno group		0 (0%)		2 (12%)	12%
Korea	Lido group	16 (6.2%)	16 (100%)	16 (6.2%)	16 (100%)	0%
	Ligno group		0 (0%)		0 (0%)	0%
	Lido + Ligno group		0 (0%)		0 (0%)	0%
Pakistan	Lido group	7 (2.7%)	6 (86%)	7 (2.7%)	4 (57%)	-29%
	Ligno group		0 (0%)		0 (0%)	0%
	Lido + Ligno group		1 (14%)		3 (43%)	29%
Thailand	Lido group	6 (2.3%)	3 (50%)	6 (2.3%)	3 (50%)	0%
	Ligno group		2 (33%)		1 (17%)	-16%
	Lido + Ligno group		1 (17%)		2 (33%)	16%
Taiwan	Lido group	4 (1.6%)	4 (100%)	4 (1.6%)	4 (100%)	0%
	Ligno group		0 (0%)		0 (0%)	0%
	Lido + Ligno group		0 (0%)		0 (0%)	0%
Singapore	Lido group	4 (1.6%)	4 (100%)	4 (1.6%)	2 (50%)	-50%
	Ligno group		0 (0%)		0 (0%)	0%
	Lido + Ligno group		0 (0%)		2 (50%)	50%
Malaysia	Lido group	2 (0.8%)	1 (50%)	2 (0.8%)	0 (0%)	-50%
	Ligno group		1 (50%)		0 (0%)	-50%
	Lido + Ligno group		0 (0%)		2 (100%)	100%

Analysis of terminology usage in Asia revealed a distinctive pattern in India, where the proportion of the Ligno group was higher in TAK and that of the Lido+Ligno group was higher in FT. In contrast, in other countries, including Japan, Korea, and Taiwan, the Lido group accounted for the highest proportion in both TAK and FT (Table 6).

Analysis of the proportion difference by country demonstrated a clear pattern in India, with negative values for the single-notation groups and positive values for the combined group. A similar pattern was observed in China, although the number of publications was limited. In contrast, no marked changes were observed across groups in Japan, Korea, and Taiwan. In other countries, similar patterns were occasionally observed; however, the magnitude of change was limited because of the small number of studies (Table 6).

Terminology Usage and Time Trends in Asia

A descriptive assessment was conducted to evaluate temporal trends in the usage of the terms “lidocaine” and “lignocaine” in Asia based on FT. Figures 2, 3 present line graphs illustrating changes in the frequency of each term over time in FT, with a comparison between Japan and India, which contributed the largest numbers of publications. For other Asian countries, the number of publications was limited; therefore, the results are presented in tabular form because line-graph interpretation would be limited.

**Figure 2:**
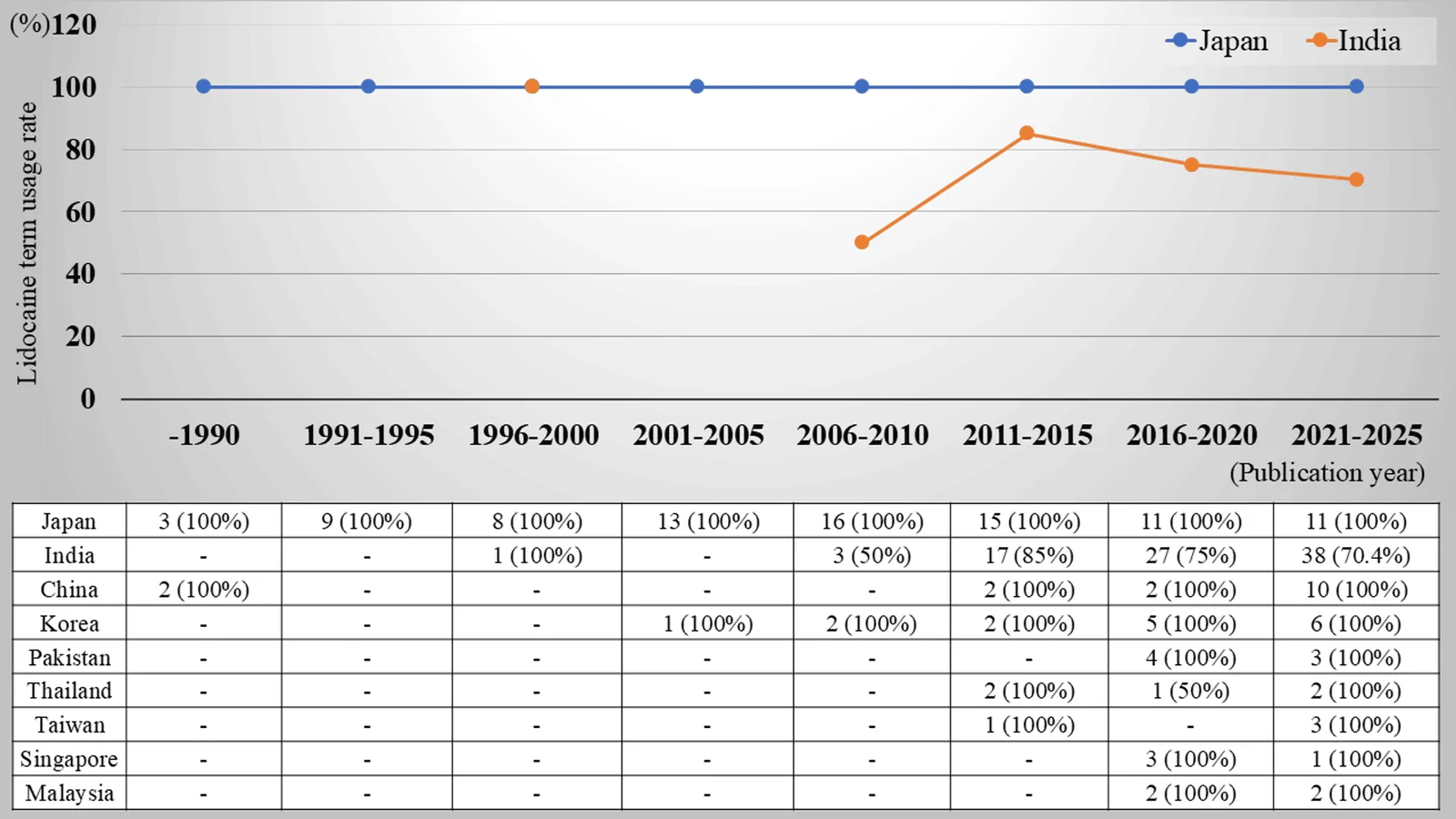
Time trends in “lidocaine” terminology in Asia The line graph shows changes over time in the proportion of articles using the term “lidocaine” in Japan and India. Country classification was based on the affiliation of the first author. The table presents the number and proportion of articles using the term “lidocaine” by country and time period, expressed as n (%). A hyphen (-) indicates that no relevant articles were identified for the corresponding country and time period.

**Figure 3:**
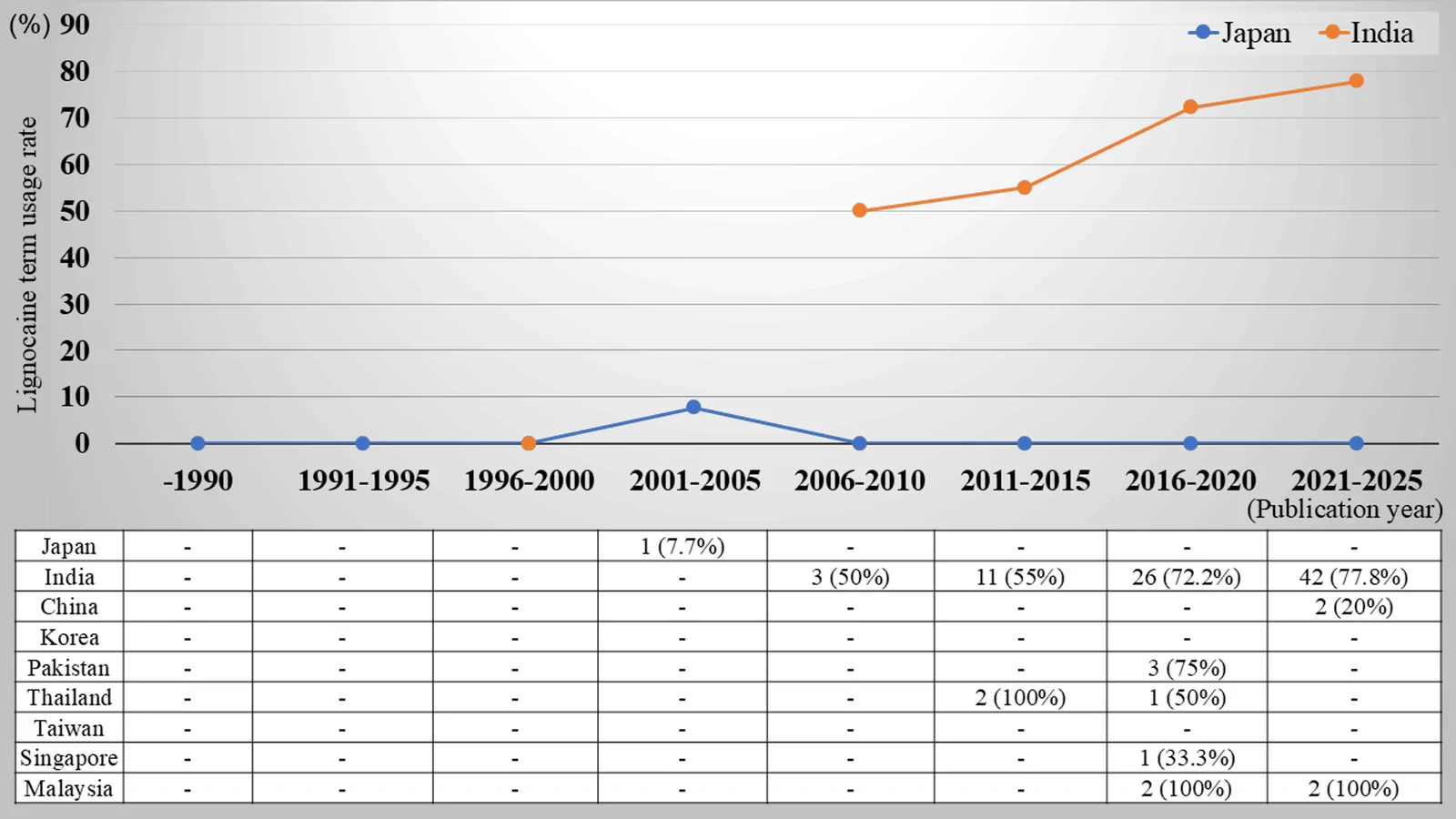
Time trends in “lignocaine” terminology in Asia The line graph shows changes over time in the proportion of articles using the term “lignocaine” in Japan and India (based on the first author's country of affiliation). The table presents the number and proportion of articles using the term “lignocaine” by country and time period, expressed as n (%). A hyphen (-) indicates that no relevant articles were identified for the corresponding country and time period.

This analysis showed that in Japan, the proportion of articles using “lidocaine” has remained consistently high since before 1990, with no marked temporal changes observed during the study period. In contrast, in India, the proportion of “lidocaine” usage decreased after 2006, whereas that of “lignocaine” increased over time.

Discussion

This scoping review systematically mapped and characterized the usage patterns of the English spellings “lidocaine” and “lignocaine” in dental academic literature by distinguishing between TAK and FT. TAK represents the bibliographic component of an article that is particularly important for readers, database indexing, and literature retrieval, whereas FT reflects terminology used throughout the main body of the publication. This distinction enabled assessment of terminology consistency within individual publications and comparison of spelling selection between bibliographic information and the corresponding full text.

The analysis was restricted to articles primarily addressing local anesthetics or local anesthesia techniques. Case reports and articles referring only to brand names were excluded because terminology use in such publications may be incidental to the principal subject of the article. This approach was intended to provide a clearer assessment of terminology selection within the academic literature on dental local anesthesia.

The proportion difference was introduced as an exploratory descriptive indicator of differences in terminology selection between TAK and FT. It was not intended to measure word-level frequency, because TAK and FT differ substantially in length and function. Rather, it provided a simple means of describing whether articles classified into each terminology group became more or less prevalent when the analysis moved from bibliographic information to the full text. Accordingly, the proportion difference should be interpreted together with the absolute number and proportion of included articles. Because terminology was classified at the article level rather than by counting individual word occurrences, the present findings should be interpreted as describing terminology usage patterns within publications rather than differences in word frequency.

Overall Trends in Terminology Usage and Inconsistency Between TAK and FT

“Lidocaine” was the predominant spelling in both TAK and FT, confirming its broad adoption in dental academic literature. However, the proportion of articles using both “lidocaine” and “lignocaine” was higher in FT than in TAK. This finding indicates that terminology presented consistently in titles, abstracts, or keywords may become mixed within the main text of the same publication.

Several mechanisms may contribute to this coexistence. Authors may retain terminology used in cited sources, particularly when discussing studies published under different regional naming conventions. In addition, manuscripts prepared by multiple authors may contain spelling variations that remain after revision. Editorial and peer-review processes may also focus more strongly on scientific content than on complete harmonization of synonymous terminology. These explanations remain hypotheses, because the present study did not directly examine manuscript preparation, citation practices, or editorial decision-making.

Terminological coexistence is not unique to dental anesthesia. Previous studies in anatomy have shown that historically derived, conventional, and standardized terms may continue to coexist in academic publications despite the availability of formal nomenclature [9]. The present findings therefore support the broader observation that standard terminology and actual terminology usage do not always correspond completely in scientific writing.

Differences Between Clinical and Basic Research

Clinical medicine accounted for a larger proportion of the included articles than Basic science. Similar predominance of clinical research has been reported in other medical fields, including urology and general internal medicine [10,11]. Therefore, the distribution observed in the present study may reflect the publication profile of medical and dental research rather than a consequence of the classification method alone.

The difference between TAK and FT was more pronounced in Clinical medicine than in Basic science. One possible explanation is that clinical studies frequently involve larger and more diverse author groups, particularly in multicenter studies and clinical trials [12]. Greater numbers of contributors may increase opportunities for inconsistent terminology to remain during manuscript preparation and revision. By contrast, smaller research teams may find it easier to maintain uniform terminology throughout a publication. However, the present study did not collect data on author number, institutional collaboration, or editorial procedures; therefore, this interpretation should be considered hypothesis-generating.

Regional Differences in Terminology Usage

“Lidocaine” remained the predominant spelling across all geographic regions. Nevertheless, the difference between TAK and FT was more marked in Asia than in other regions. Because Asia also contributed the largest number of publications, this observation should not be interpreted independently of regional sample size.

Regional variation in terminology may reflect differences in historical language use, educational background, journal style, and publication practices. Previous studies have reported geographic patterns in publication and citation behavior, including increasing contributions from countries outside traditional English-speaking publication centers [13]. Such developments may increase the diversity of English terminology used in international academic publications.

Europe and North America showed smaller differences between TAK and FT. This may be associated with relatively stable journal conventions and established preferences for particular drug names. However, the present data do not directly demonstrate that editorial practice or writing culture caused the observed regional patterns. These factors should therefore be regarded as possible explanations requiring further investigation.

Country-Specific Patterns in Asia

India contributed the largest number of publications within Asia and showed the most distinctive difference between TAK and FT. The Ligno group was relatively prominent in TAK, whereas the Lido+Ligno group was more frequent in FT. In contrast, Japan, Korea, and Taiwan predominantly used “lidocaine” in both components and showed comparatively limited differences between TAK and FT.

The FT-based temporal assessment also indicated contrasting patterns between Japan and India. In Japan, “lidocaine” remained the predominant spelling throughout the study period. In India, the relative use of “lignocaine” increased after 2006, whereas the use of “lidocaine” decreased. These findings describe changes in published terminology but do not establish the reasons for those changes.

Several recent Indian clinical studies have compared 2% lidocaine with other local anesthetics in dental practice [14-17]. The accumulation of such publications may have reinforced the continued use of terminology commonly adopted in Indian dental literature. However, the increase in the Lido+Ligno group should not be interpreted as evidence of a change in clinical drug selection. It more likely reflects the coexistence of synonymous names within individual articles, although citation patterns, journal style, and author preference were not directly examined.

The relative consistency observed in Japan, Korea, and Taiwan may suggest more stable preferences for the term “lidocaine.” Nevertheless, the number of publications from Korea and Taiwan was limited, and comparisons among countries should therefore be interpreted cautiously.

Potential Implications of Terminology Inconsistency

Terminological inconsistency has potential implications beyond manuscript style. In principle, literature searches using only “lidocaine” or only “lignocaine” could fail to retrieve relevant studies, particularly when search strategies do not incorporate synonymous terms. Such inconsistency therefore has the potential to affect the comprehensiveness and reproducibility of systematic reviews, scoping reviews, and other forms of evidence synthesis.

The issue may also be relevant to database indexing and automated literature processing. Although databases often map synonyms through controlled vocabularies, terminology appearing in titles, abstracts, keywords, and full text could potentially influence retrieval performance. Likewise, AI-assisted literature searches, natural-language processing systems, and automated evidence-screening tools may be influenced when synonymous drug names are used inconsistently or when training data disproportionately reflect one regional spelling.

However, the present study did not directly evaluate literature retrieval performance, database indexing, evidence synthesis, or AI-assisted search systems. Therefore, these potential implications should be regarded as hypotheses derived from the observed terminology patterns and should be verified in future studies.

Several approaches may reduce these potential problems. Authors could select one preferred term and use it consistently throughout the manuscript while introducing the alternative spelling at first mention. Journals could also incorporate terminology checks into editorial style guidance, and literature search strategies should routinely include both spellings. Such approaches may improve discoverability, facilitate international academic communication, and reduce avoidable ambiguity in dental and medical literature.

Research Gaps and Future Directions

The mapping conducted in the present scoping review highlighted several areas for further investigation. First, the available literature was unevenly distributed across geographic regions, indicating that terminology usage remains less well characterized in some regions and countries. Second, the potential effects of inconsistent use of “lidocaine” and “lignocaine” on literature retrieval, database indexing, and automated literature processing remain to be directly investigated. Future studies addressing these areas may provide a broader understanding of the implications of synonymous terminology usage in dental academic literature.

Limitations

This study has several limitations. First, it was a descriptive analysis and did not examine causal relationships between terminology usage and citation practices, authorship structure, editorial policy, educational background, or regional writing conventions. Explanations proposed for the observed patterns should therefore be interpreted as hypotheses.

Second, the literature search was limited to PubMed and Web of Science and to English-language publications. Relevant articles indexed elsewhere or published in other languages may not have been captured. In addition, the focused search strategy using the term “dental” may not have identified all relevant dental articles that did not contain this term in the searched records. The regional distribution of the included literature may also have influenced the observed results.

Third, inclusion required the presence of at least one of the two spellings in both TAK and FT. This criterion was necessary for within-publication comparison but excluded articles in which the terminology appeared in only one component. Consequently, the full diversity of terminology usage in dental literature may have been underestimated.

Fourth, case reports and articles referring only to brand names were excluded. Terminology usage in publications closely reflecting routine clinical practice was therefore not evaluated.

Fifth, the proportion difference was introduced as an exploratory descriptive indicator and has not been externally validated. Because it represents differences in article-level terminology usage between TAK and FT, it should not be interpreted as a direct comparison of word frequency. Furthermore, full-text articles generally contain substantially more words than titles, abstracts, and keywords, which may increase the likelihood that both terms appear within the same article. Therefore, part of the observed increase in concurrent use of “lidocaine” and “lignocaine” in FT may reflect differences in document length rather than terminology preference alone. Future studies should evaluate terminology usage using normalized measures, such as term frequency relative to total word count, to better account for differences in document length.

Finally, no inferential statistical comparisons were performed. Accordingly, the present findings should be interpreted as descriptive observations of terminology usage within the included literature and should not be considered evidence of causal or mechanistic differences among specialties, regions, or time periods.

Despite these limitations, this study provides a structured assessment of terminology consistency by distinguishing between bibliographic information and full text. The analytical framework may also be applicable to investigations of other synonymous drug names and domain-specific terminology. The present findings provide baseline descriptive data that may inform future studies on terminology usage and standardization in the dental literature.

## Conclusions

This scoping review systematically mapped the usage patterns of the English spellings “lidocaine” and “lignocaine” in English-language dental academic literature by distinguishing between TAK and FT. Although “lidocaine” remained the predominant spelling, coexistence of the two synonymous terms within individual publications was common, particularly in FT. Differences in terminology usage based on the country or region of the first author's affiliation were also observed; while papers by first authors affiliated with Indian institutions exhibited distinctive patterns, terminology usage was relatively consistent in papers by first authors affiliated with institutions in Japan, South Korea, and Taiwan. These findings provide descriptive information regarding terminology usage in dental academic literature and may serve as baseline data for future investigations of terminology standardization. The analytical framework presented in this study may also be applicable to investigations of other synonymous medical and dental terms.

## Appendices

Supplemental data

The complete list of the 526 articles included in this scoping review is available as supplemental data via Zenodo: https://doi.org/10.5281/zenodo.22020518
